# Practical Observations on Disorders of the Stomach, with Remarks on the Use of the Bile in Promoting Digestion

**Published:** 1810-09

**Authors:** 


### CRITICAL ANALYSIS
OF THE
### RECENT PUBLICATIONS
IN THE
### DIFFERENT BRANCHES OF PHYSIC, SURGERY, AND MEDICAL PHILOSOPHY.

---

*Practical Observations on Disorders of the Stomach, with Remarks on the Use of the Bile in promoting Digestion;* by GEORGE REES, M. D. Member of the Royal College of Physicians, senior Physician to the London Dispensary, &c. 8vo. pp. 199. London, 1810.

THE importance of the organs and functions which are the subject of the present work, has been acknowledged in all ages; and the increase of luxury and refinement among the opulent; of the abuse of spirituous liquors among the lower orders, with some other causes not necessary to be specified here, have increased the frequency of disorders in the chylopoetic viscera in a rapid progression. For these reasons, most probably, the author's attention was first turned to the investigation of this highly important subject. These Practical Observations are preceded by the anatomy tomy and physiology of these organs, on which it is unnecessary for us to remark, as there is nothing materially different from the best writers on the same subject. On some occasions, however, Dr. R. seems disposed to provoke criticism, as when he asserts that the operation of many medicines is confined to the stomach *alone*; which opinion he supports by instancing the effects of diffusive stimulants only.

"I believe," he says, "there is a certain definite quantity of nervous fluid, or nervous energy, which every constitution is capable of supplying; this, in the natural state of the animal body, is regularly diffused throughout every part, in a degree proportionate to the functions which that part has to perform.

"Wherever, therefore, there is any preternatural accumulation of this fluid, though that part is capable of extraordinary exertions, the rest of the system is impaired. Observation confirms this remark, for those who have at a very early period of life evinced uncommon powers of understanding, have generally suffered from epileptic fits, or died prematurely.

"As instances of this may be mentioned, Mozart, the late amiable Kirk White, Mr. Gilpin's son, and many others.

"Hence there is some foundation for a common popular expression, that a child is too clever to live; this being, like most other proverbs, founded on experience.

This principle will aid us very materially in explaining the peculiarities of disease, and especially the actions of the Stomach; for every thinking person must admit, that the operation of the vital principle must be resorted to, in order to explain the actions of a living body.

"This definite quantity of nervous energy in the system, and its irregular and preternatural determination to particular parts to the detriment of other organs, is one opinion which I am *solicitous to maintain*.

"Intense application of the mind, by diverting the energies from the stomach, render it less capable of action.

"A person may sit down to dinner in good health, and with an eager appetite, but the sudden reception of some distressing intelligence may instantly destroy the inclination for food, or occasion its being rejected if swallowed. To a certain degree, therefore, appetite may be regulated by reason.

"It deserves to be noticed, that this controul over the appetite is never met with but in persons of a strong constitution; and there is perhaps no better method of ascertaining the strength of the system, than by observing the effect of abstinence, and knowing how long it can be endured.

"Where habit has not thus gained an ascendancy over appetite, the sight of food occasions an immediate secretion of nervous fluid: hence the expression, *the mouth waters*, which is perceptibly realized in dogs, who discharge a considerable quantity of saliva when stimulated by the sight of food after long fasting.

"The

"The phænomena of extreme hunger do not very frequently present themselves to our view, and therefore I shall be excused for introducing here a very interesting narrative, that strikingly exemplifies the doctrine now laid down.

"I have talked," says the author, "with the captain of a ship, who was one of six that endured it in its extremity, and who was the only person that had not lost his senses when they received accidental relief."

"He assured me that his pains at first were so great, as to be often tempted to eat a part of one of the men who died, and which the rest of the crew actually for some time lived upon.

"He said, that during the continuance of this paroxysm, he found his pains insupportable, and was desirous at one time of anticipating that death which he thought inevitable, but his pains, he said, gradually decreased after the sixth day, (for they had water in the ship, which kept them alive so long) and then he was in a state rather of languor than desire; nor did he much wish for food, except when he saw others eating, and that for a while revived his appetite, though with diminished importunity. The latter part of the time, when his health was almost destroyed, a thousand strange images rose upon his mind, and every one of his senses began to bring him wrong information.

"Perfumes appeared to him to have a fœtid smell, and every thing he looked at had a greenish hue, and sometimes a yellow. When he was presented with food by the ship's company, who took him and his men up, four of whom died shortly after, he could not help looking upon it with loathing instead of desire, and it was not till after some days that his stomach was brought to its natural tone, when *the violence of his appetite returned with a sort of canine eagerness.*" *

On the important subject of Digestion, Dr. R. appears to be solicitous to establish his own definition, viz.

"*Digestion is that process, by which the vitality of the food is separated from the substance with which it is combined.*

"In offering this definition, I am sensible that I lay myself open to animadversion; and if those who take upon them to examine, will combine candour with criticism, I can have no objection to the investigation of the opinion. Had I substituted the words *nutritious principle* for vitality, I should not have been amenable to censure; but the question relative to the nature of food, and its power of replenishing the exhausted strength of the system, would have remained unanswered, whilst the term I have now adopted, conveys some intelligible idea; and though not in its nature susceptible of experimenral proof, furnishes a rational and satisfactory explanation of digestion and its effects.

"That all animals, possessing the power and exercising the functions

" * Vide Goldsmith's History of the Earth, vol. ii. p. 116.

functions of muscular motion, are exhausted by fatigue, and require to be renovated by food, is a fact too palpable to be denied; and it is therefore only necessary to discover in what manner food is capable of imparting this vigour to the system.

"Now, as it is self-evident that what is lost must be restored, we are drawn into a confession either that the process of digestion is capable of forming, by a peculiar and *unintelligible combination of matter*, some substance capable of repairing the loss of strength and energy, or it acts by separating it from the materials subjected to its operation; and this last is the opinion I maintain."

Although we can have no objection to this last expression, "*a peculiar and unintelligible combination of matter*," as forming a part of the definition of digestion; yet we are unable to comprehend how *any process* can separate *vitality* from dead matter, which contains no vitality. As we think it fair in criticism to attempt substituting something less exceptionable than what is objected to, we would define digestion to be that process by which *dead matter is converted into living nourishment.* We agree with our author, that the process by which this is accomplished, may most probably be a peculiar and unintelligible *combination* of matter. The minute discussion of this single question would require a volume. The author concludes the subject of digestion in the following summary.

"From what has been advanced, it is obvious that the appetite for food is a natural instinct which ought to be consulted rather than directed.

"It is an instinct which, independent of reason, teaches us what to renounce, and what to prefer; and we seldom eat what is pernicious, without feeling an inward conviction that the stomach does not approve it; hence this organ has been emphatically denominated *the Conscience of the Body.*

"The first impression of putrid food is made on the sensorium through the medium of the olfactory nerves; and it is a curious circumstance, that in all animals the nose is placed in close proximity to the mouth, undoubtedly for the purpose of bringing the food under the examination of this sense.

"I have detailed these facts relative to digestion as illustrative of the truth of the position, that

"*Digestion is that process, by which the vitality of the food is separated from the substance with which it is combined.*

"But as a variety of secretions are provided by nature for the completion of this important function, I now proceed to the consideration of their properties; and first, of the Saliva."

Besides the commonly acknowledged offices of the saliva in moistening the dry food during mastication, and aiding the process of digestion, our author assigns it another function, which is far from being generally acknowledged. This is the power which the salivary glands possess as excretory organs, capable of carrying ing excrementitious and noxious matter out of the system. In confirmation of this opinion, Dr. R. gives the following facts.

"A lady whom I am well acquainted with, lay-in of her second child, and experienced no particular difficulty in her labour; she is of a delicate constitution, and had been subject, on slight irregularities, to a discharge of the fluor albus.

"For two or three days after her confinement she continued very well, and had a free discharge of the lochia. She was recommended to take bark and other astringents, but whether from these, or what is more probable, from accidentally taking cold, the lochia was suddenly checked, the head became affected with stupor and giddiness, and the face swelled.

"This was presently followed by a very copious salivation, which continued for several days. Opening medicines were now recommended, and relaxants at night; the lochia was thus brought on, and salivation immediately disappeared.

"A young man consulted me a short time ago, whom I visited in company with Mr. Evans, Surgeon in Old Street; he said he had had a chancre on the penis six months before, and used mercury then for a few weeks *only*, but not to such a degree as to produce any sensible effect upon the mouth.

"On examining the inside of the fauces, a very general and deep-seated ulceration was observable on the uvula and tonsil glands, and a profuse salivation to the amount of *two quarts a day* attended it, without the least mercury as he positively assured us, having been used since the above-mentioned period. As this gentleman's constitution appeared to be considerably enfeebled, and his habit scrophulous, we thought it adviseable to give him first a gentle emetic, and afterwards bark: we adopted this plan, but without effect, the spitting still continuing, and the ulceration extending its ravages. We now employed mercurial ointment in the usual manner, and after rubbing in on the thigh two drachms of mercurial ointment for three nights, the *spitting considerably diminished*, and the ulceration rapidly healed; after this time the spitting continued in such a moderate degree as might naturally be expected to arise from the ointment itself.

"From these and many similar cases which occasionally occur we may conclude, that the saliva frequently becomes an excrementitious fluid, conveying something out of the body which would prove noxious if it were retained; and thus we may on some occasions recommend the smoaking of tobacco."

The offices of the *Bile* are more numerous and apparently more important than those of the saliva. Its being secreted from venous blood instead of arterial, would induce us to admit, that it is in a certain degree an excrementitious fluid. That it contributes 'by its action on the intestines, to promote the expulsion of the fæces out of the body,' appears to be generally acknowledged. But its office in promoting and perfecting the important process of digestion, and its being a principal cause of the sensation of hunger, are not so generally known or admitted. These two points Dr. R. is anxious to establish on a solid foundation, "and that from the defect, not the redundance of bile, the most pernicious consequences ensue, I trust," he says, "I shall be able to prove to the satisfaction of those, whose understandings are not warped by prejudice.

"I should be entering into the regions of theory, were I to attempt an explanation of the change which the blood undergoes previous to its entering the vena portarum; and as the doctrine I now propagate relative to the use of bile in digestion, is of itself liable to opposition, I think it prudent for the present to suppress what I conceive to be a feasible opinion on this head.

"I proceed therefore to maintain, that the bile is an important agent in digestion; and to prove this, I may in the first place mention, that *whenever the secretion of this fluid is diminished, the functions of the stomach and the process of digestion are deranged.*

"I shall adduce a Case, not from any remarkable peculiarities attending it, but merely as descriptive of my meaning: it is such as every medical practitioner must have frequently seen, and therefore more valuable as an illustration, since it enables me to convince the reader by a reference to his own experience.

"M. D. aged 55, or thereabouts, complained of a short dry cough for several months, with entire loss of appetite for nearly the same period; the countenance looked somewhat emaciated, but had no particular sallowness; and the urine, though deficient in quantity, exhibited no morbid appearance.

"This patient (a female) was very much troubled with flatulence, and when I saw her was much distended by a dropsy of the abdomen, and anasarcous swelling of the legs; she had drank considerable quantities of spirituous liquors, and this had impaired the constitution.

"I omit, for the sake of brevity, the detail of the treatment, which did not of course prove successful; the woman died; her body was examined after death, and it was found that the whole substance of the liver was knotted and tubercular, extremely hard, and of a whitish colour; not the least bile was found, and no traces of a gall-bladder could be discovered. This is a peculiarity not commonly met with. The stomach was cut open and carefully examined, but presented no appearance of disease.

"Here is one instance, then, of impaired appetite from defective bile; in this case the whole substance of the liver was so completely indurated, that nothing like bile could be formed, and this probably was the reason that the countenance exhibited no jaundiced appearance.

"Whenever we perceive jaundice in the skin, or in the eyes, when the yellow colour is very predominant, we may be assured the liver is capable of forming bile, and that it has been first formed, and then absorbed; it accords with my experience therefore fore to state, that in general these are the most curable cases, and arise from causes more manageable and accidental.

"Without any discolouration of the skin, the liver may be very much impaired both in function and structure, and dropsy take place in consequence: in proof of this I could adduce some very interesting cases, which have come under my observation, were it not digressing too much from the object before me. As far as one fact can tend to corroborate an opinion, this which I have now mentioned is conclusive, there being no apparent fault in the stomach, but entire loss of appetite, from the total absence of bile.

"That it proceeds from this, and not from any sympathy between the stomach and the liver, is certain, because the obstruction to the passage of the bile by a gall-stone, without any disease in the liver itself, produces a similar effect.

"It is fortunate for the support of my opinion, that what I now advance is not confined to my own personal experience; and I appeal to every one who reads and practices for the confirmation of what I say: if, therefore, it be true that the absence of bile does impair the digestion, the converse of the proposition is most likely to be true also.

"When a person in maintaining an opinion, draws an inference from something which once happened, but which can no longer be repeated, the ground of his arguments may very fairly be called in question.

"Instead of attempting to inveigle the approbation of the reader in this manner, I recommend him to examine the truth and accuracy of my assertions by a comparison with his own experience; and having asserted the absence of bile to be the great cause of indigestion, I submit the following proposition to the same test of examination, that *bile in the stomach is a very powerful stimulus to hunger.*

"I suspect this paragragh will strike the reader as very heterodoxical, and he may perhaps exclaim, How can this be true? do we not often meet with cases, where people have every appearance of being bilious, in which loss of appetite, nausea, and vomiting, are the predominant symptoms? and is not the tongue white, and covered with a kind of whitish crust? In addition to these, does it not often happen, that large quantities of bile are thrown off from the stomach, to the immediate and effectual relief of the patient?

"These are very reasonable interrogatories, and such as I feel it incumbent on me to explain, if I expect to gain proselytes to my opinions. I begin with the first question, by considering what are the symptoms, from which we are led to say a patient is bilious.

"They are these: a sallow complexion, yellowness in the white of the eye, and a little tinge of the same colour around the temples; great languor and lassitude, a disrelish of animal food, and a partiality for vegetables of the acescent kind, drowsiness, giddiness, and a white tongue.

"Some or more of these are generally found to accompany the loss of appetite, nausea, &c. already alluded to. Now all these symptoms I most decidedly affirm, originate, not from the presence of bile in the stomach, but from its absence, and from its retention in the circulation. This I think cannot reasonably be doubted by any person who will take the trouble to reflect upon the subject; and of those who do doubt, it will be found that their practice is in opposition to their opinions, for it is agreed, that the most effectual means of relieving these are vomiting and purging. We give calomel to emulge, as it is often expressed, the biliary ducts; what is the effect of this, but to encourage the discharge of bile from the liver into the duodenum? But the symptom which most frequently gives rise to erroneous conclusions relative to this point, is the appearance of the tongue.

"*A white tongue.* When a person is attacked with fever, the surface of the tongue very frequently exhibits a morbid appearance, in some being white, in those of the more malignant kind, or towards the decline of the disease, brown, and sometimes black: hence many are disposed to consider this change as an indication of fever; and so prevalent is the notion among the public in general, that when you ask to see the tongue, the answer frequently is, I have no fever.

"But this state of the tongue is merely accidental; it is by no means essential to fever, since it is very often found where fever does not attend; and frequently in the most severe attacks of that disease, the tongue exhibits no discolouration at all.

"I know a person who has for several years been engaged in a large white lead manufactory; his appetite has long been imperfect, attended with constipation of the bowels, and his tongue continually covered with a complete whitish incrustation, but without either fever or thirst. This variation in the appearance of the tongue may be easily explained, for as fever may attack a person under different states of the system, it is that state of the system, and not the fever itself, on which the appearance of the tongue will depend.

"But the error to which I now make an allusion is this, that when the tongue is white, the patient is supposed to have bile in the stomach; this I have repeatedly witnessed, and have seen calomel, rhubarb, jalap, &c. often recommended with the *professed intention* of carrying the bile out of the stomach; and I avail myself of this point to convey the distinction between my own sentiments and that which I believe to be the general one. I say, that in this case, the whiteness of the tongue proceeds from the *deficiency of bile* in the primæ viæ.

"The facts which I shall presently adduce will bear me out in this this declaration, and therefore I proceed to explain what certainly seems to be the most plausible objection to the doctrine, *that large quantities of bile are thrown off from the stomach, to the immediate and effectual relief of the patient.*"

As these opinions of our author are in a great degree peculiar to him, and, if well founded, of great practical importance, we have given his own statement of them, that there may be no misrepresentation. The last objection, the plausibility of which he admits, receives a very full and ingenious answer, supported by facts and cases, for which we must refer our readers to the work itself.

When Dr. R. has established his new opinions on what we think a very fair basis, he proceeds to draw practical inferences from them. When the bile becomes deficient, he reminds practitioners of what has long been known and employed; the substitution of artificial bile or else that of other animals. As bile is an animal soap which has been analysed, all the constituent parts except the bitter principle are understood. The origin and formation of this principle in an animal secretion, when the animal never tastes any thing bitter, will probably always remain among those mysterious processes, by which animals and vegetables are found to *create* substances which their own bodies or nourishment never were found to contain. The following extract from Van Swieten's Commentaries, appears very apposite to this subject.

"The bile and phlegm are of so opposite a nature, that they can never predominate together, bile being the greatest detergent, dissolvent, and attenuant of all pituitous matter. If the bile be hindered from flowing into the duodenum, and by this means be thrown back into the blood, it dissolves it to such a degree, that after a long jaundice there usually follows a dropsy. Whenever this viscid, pituitous matter is accumulated, the bile is either deficient in quantity, or it is too inactive. Nothing, therefore, seems more proper in this case, than to supply the defect of the bile, either by giving the bile of some other animal, or by the use of bitter plants, such as wormwood, centaury, &c. The former seems the most natural method, and for this reason the bile of the most voracious animals that use no manducation, nor have several of the other aids of digestion, has been chosen principally for this purpose, as in these a sharper bile than ordinary seems to have supplied the want of the other. Thus the gall of a jack, that devours fishes whole, and of eels, has been much commended for this purpose.

"Zoographers observe, that the fiercest animals have the most acrid bile, and for this reason, the apothecaries keep in their shops the gall of bulls inspissated; and perhaps that most costly porcupine stone, called perdro del porco, may owe its virtue, as well as its original, to bile."

Here then theory and practice correspond, and a wide field is open for the physiologist and experimentalist, which, if properly cultivated, promises to be productive of the best effects.

 Dr. R.

Dr. R. having finished his discussion of the uses and importance of the bile, in regulating the appetite and promoting digestion, he adopts the common opinion respecting the pancreas, and proceeds to the signs of a *weak stomach*. These he treats of in considerable detail; the first of them is a *florid countenance*; and he takes care to distinguish, by their proper signs, the florid hues of rural health from the morbid efflorescence which indicates the feeble state of the stomach; and very properly distinguishes between that appearance which is the effect of hard drinking, and that which accompanies the most perfect temperance. In the treatment of this state of the stomach, Dr. R. suggests some cautions which we think important.

"Great circumspection is necessary in the management of those who have the complexion now described; their peculiarities with respect to diet ought to be carefully enquired into; for even a draught of cold water to such persons would at times prove a poison.

"Wine, especially port, generally turns acid: all eruptions in such persons, however trifling, are critical and constitutional, and should never be repelled.

"Bleeding, under any circumstances, in these habits cannot be resorted to without danger: even medicines of moderate activity, must be given in very small doses. The neutral salts, unless combined with warm carminatives, disagree, producing spasms, and severe griping. I know some persons, for whom ten grains of magnesia is a sufficient dose to procure three or four stools, and this is found frequently to answer the purpose of an aperient better than any other.

"This may be owing to the acidity of the stomach, with which such patients are frequently troubled; and indeed it appears to me that the foundation of the complaint is the want of a proper secretion of bile into that organ.

"I know a lady who was recommended to take half a drachm of rhubarb, which was made into eight pills; by mistaking the directions, she fortunately only took one, and this operated briskly; so that in this, as in other similar cases, an ordinary dose of medicine would be a dangerous remedy; the warm tinctures, those of rhubarb, senna, and aloes agree best; oily medicines are in general very obnoxious, and the common neutral salts too cold; they produce great oppression at the stomach, violent sickness, fainting, and spasms. Nothing is more common than to hear these patients declare, they thought they should have died from taking them.

The next indication of a weak stomach, is the "inability to continue long without food." Those who labour under this infirmity are advised to dine early, and not to suffer that inward sinking, or sick head-ache, to continue or supervene by long fasting. He considers nervous head-achs and hypochondriasis as indications of a weak state of the stomach; oppression, and heaviness after dinner, and flatulence, which being very often constitutional, admit of only only temporary relief. He enumerates some other signs of this infirmity, which seems more equivocal, such as the following three.

"1. A sense of langour and lassitude suddenly coming on the lower limbs.

"2. A frequent desire to make water, especially on any slight agitation of mind, and the evacuation of pellucid urine without smell.

"3. In women, large full breasts, or rather breasts surrounded with a very large proportion of fat, denote the same thing.

"4 The use of spirituous liquors, even in small quantities, proving highly prejudicial."

He thinks that mothers who suckle too long, frequently induce these disorders of the stomach.

"This is an error that ought to be corrected; for when the practice of suckling is continued much beyond the powers of the system, it brings on a train of incurable evils.

"What that change is which is thus produced it is exceedingly difficult to define; suffice it to say, it is such as no future amendment of the health will remove. Spasms of the stomach, flatulencies, epilepsies, and particularly the sick nervous head-ach, are thus engendered in the habit.

"If she feel a great and unusual sinking inwardly, sighs very often, looks pale and *hollow-eyed*, with a dark circle under the eye-lids; if she have giddiness and vertigo, with occasionally temporary loss of sight, a little short dry cough, a slight oppressive pain about the chest, then there can be no question, either that she has suckled too long, or is disqualified for discharging the duties of a nurse.

"Nervous head-achs in men, comparatively, but rarely occur; they are alike indications of a weak stomach, and return periodically; go off spontaneously after continuing six or eight hours, and leave some weeks interval of perfect ease. These intervals of ease are the strongest marks of distinction between this and head-achs arising from other causes.

"There is scarcely a point in practice that requires nicer discrimination, than the different species of head-ach; they often assume appearances very similar to nervous affections, when they originate, not from debility, but plethora.

"In the former instance, they very generally arise from the stomach, and are to be relieved through the medium of that organ; in the latter, the deranged functions of the stomach are attributable to the alteration in the head."

Dr. R. then subjoins a number of judicious and valuable observations respecting the various causes and treatment of the different kinds of head-achs, which we particularly recommend to the attention of junior practitioners.

On the very frequent and distressing symptom denominated flatulence, Dr. R. gives the following directions, and reference to a case where *bleeding* was found an effectual remedy.

 "From

"From the general complexion of the patient, her age, her strength, and her peculiar situation, bleeding was recommended, which gave great and immediate relief, and the use of aloetic medicines, with relaxants occasionally, completed the cure.

"This case is not unfrequently met with; but, excepting in these particular circumstances, few practitioners would think of employing the lancet to relieve flatulence at the stomach, which in the cases where it generally occurs would be aggravated by the debility it is calculated to induce. The propriety of this remedy will be indicated by attending to the following circumstances.

"First, Constitutional character, the general health, and the appearance of plethora.

"Second, occasional palpitation of the heart, especially if in a warm room.

"Third, The age of the patient, puberty especially.

"Fourth, The flatulence being relieved rather than increased by fasting; this is, perhaps, the most decisive.

"The juvantia and lædentia, which often give more information than any thing else; if strengthening and tonic remedies do harm, the contra indications are obvious.

"Sixth, In women menstruation never having occurred, or its being suppressed: and,

"Seventh, and lastly, The suppression of any habitual evacuation. These circumstances, taken collectively, will be sufficient to direct our practice."

These rules are followed by some valuable observations on the causes and prevention of flatulence, to which the annexed case is subjoined.

"The following case I copy verbatim from my note-book.— It shews several striking peculiarities, and in some respect confirms the fact, that certain persons have had the power of returning their food like ruminating animals.*

"*Case of Flatulence, with the Regurgitation of Food, and the Method of Cure.*

"Eliz. Wilbraham, æt. 45, married, and has had children; when 12 years old caught a fever, which continued three months, and affected her brain very much; since that time (but certainly not before) she has had a very weak stomach; what is remarkable, she cannot digest beef; whenever she has eaten it, it always produced great uneasiness and a sense of weight and pain at the stomach, which continued about half an hour, and then was rejected bit

* Dr. Arbuthnot, in his Treatise on Aliments, says, there have been several instances of ruminating men; and that quality leaving them was a symptom of approaching sickness. Vid. Arbuthnot on Aliments, p. 222. For illustrations of this fact, vid. Philos. Trans. & Bonetus's Sepulchret.

bit by bit by belching with wind (not with retching and vomiting) till the whole seemed to be returned.

"This is not the case with any other meat, and though the fat of any meat is very obnoxious to the stomach, it does not come up like beef.

"All vegetables agree with her except potatoes; every kind of meat eaten hot produces a great sense of distention, but cold meat does not.

"Tea seems to agree tolerably well; she never finds any acid or heart-burn, but has often a sense of fluttering like a bird at the stomach.

"Walking quick always brings on violent flatulent eructations, which continue an hour or more without intermission; she once tried, by the advice of a friend to suppress it, but severely repented the experiment, as she thought it would have killed her; her menses have always been regular, but her symptoms are generally worse a day or two before they make their appearance; the bowels are regular.

"Though this patient is positive that the fever impaired the functions of the stomach, the violent flatulence has continued only five years, and for this she knows no other cause than continual sitting at the needle. It first began with pain in the stomach, which seized her in one moment, and darted through the left side; this at first only three or four times a week, attended with the globus hystericus in the throat to such a degree, that she could neither speak nor swallow.

"These symptoms always came on between twelve and one at night, and have continued, with only a few days intermission, during the period already mentioned. I should have observed, in detailing the peculiarities of this case, that the stools have always been of a natural colour.

"No medicines, however palatable, will remain on the stomach unless given in the form of pills."

The particular treatment of this unusual case is given in detail, and merits the attention of practitioners, though we do not hold it up as a model. Our author concludes this part of his observations on flatulence in these terms.

"I believe, in most cases, flatulence proceeds not from the extrication of air from any substance taken into the stomach, but is secreted from the stomach itself. In the case above described, any agitation of the body would bring on flatulent belchings to a great degree, so would a glass of wine, when no food had been taken for some time; and as the quantity thus evolved could not possibly be contained in its æriform state in the stomach, so neither is it probable that it arose from the food which had probably been digested before, and which would be assisted rather than retarded by the exhibition of this cordial.

"This subject appears to me to be very little understood, and to merit further inquiry."

He

He next examines the connexion between flatulence, phthisis, and the cessation of the menses, and gives very appropriate directions and cases. He at length arrives at what our readers may probably have expected much earlier, *viz.*

"*Treatment best calculated to strengthen the Stomach.*

"As the cure of a disease is the great object of practice, this part of the subject may be considered the most interesting, and it appears to me the most difficult to describe, for nothing can be advanced but with relation to particular circumstances, on which the judgment and discrimination of the practitioner must after all be called upon to decide.

"This is obviously a difficulty inseparable from the subject, and will therefore, I trust, plead an excuse for itself. The remedies for a weak stomach are not to be found in bark or bitters solely, though they may contribute a share; they must be aided in their operation by an attention to diet, drink, exercise, cloathing, &c. points of most essential consideration for the valetudinarian of this description."

*Diet.*—The importance of this subject has always been confessed, and its rules are few and simple enough, but the application of them to particular cases is extremely difficult. For no two stomachs are precisely alike, nor the same stomach at different times. "There is, says Dr. R. a radical difference in the digestive organs from the first moment of conception; and it should be the great object of a practitioner to find out the constitutional difference. This can only be acquired by careful observation, and hence, the necessity of early and vigilant attention to disease."

The patient's own feelings and experience, if he is sufficiently attentive to them, will furnish the best rules of diet. The common rules are better known than practised. No one requires to be told, that the quantity and quality of the food should be proportioned to the labour or exercise in the open air; that spirits are injurious; that animal food is more nutritious and stimulant than vegetable, and therefore not so suitable to sedentary pursuits as to laborious exercise; or, that a due mixture of both is better than either alone. Some of Dr. R's rules of diet seem to push abstemiousness farther than present habits and manners will be disposed to follow him, without many exceptions; as, when he says, "animal food once a day is generally sufficient, a glass of wine or half a pint of porter, may be used with this meal to warm and strengthen the stomach, but on this principle alone, not as having themselves any nourishment at all. The necessity either of wine or porter is the effect of a weak habit, or of early indulgence, and should, if possible, be avoided.

"In London, the common people are accustomed to consider porter an essential part of their support; but parents should be very very careful not to let young children drink any thing but water, for all that is naturally required from drink is to dilute the food, not to stimulate the stomach."

On the subject of *Medicines*, Dr. R. dwells particularly on the proper employment of emetics, and a proper choice of them. His advice and observations are thus summed up.

"It is a common idea, that the intention of an emetic is merely to clear the stomach; and as this is not always necessary when it is recommended, it is not easy to convince the patient of the propriety of the practice; but this is the least advantage attending its use.

"Vomiting, while it has the effect certainly of relieving the stomach of its contents, tends likewise to emulge the biliary ducts, to increase the secretions of the gastric and pancreatic juices, and to diffuse an energy to every part of the system: it is, therefore, often advisable to administer it when there is no reason to suspect any foulness in the stomach.

"In cases of dyspepsia, the benefit it imparts is probably to be attributed principally to its power of increasing these secretions, as they chiefly contribute to the process of digestion.

"An emetic may be safely given and repeated in the most delicate habits, and for the relief of a weak stomach is advisable, as a prelude to other medicines, especially if there is any reason to suppose an acid in the stomach, which is to be judged of from the heart-burn, flushing, and redness in the face, and hiccup. This acid arises from a defective secretion of bile."

"*Of Pain in the Region of the Stomach.*

"On entering, says our author, into the investigation of these complaints, the first circumstance to be observed is this, that a pain referable to that part is no proof at all that the stomach is the organ to which our remedies should be directed. Such a precipitate conclusion has been productive of considerable mischief, and therefore I wish strenuously to caution the reader against so hasty a decision.

"When a person complains of pain in the stomach, the first material question to be ascertained is, what is the cause? taking it for granted that the stomach is only acting a secondary part."

He then mentions as causes, irregularity in females, stone in the kidneys, stricture, suppression of habitual evacuations of any kind, inflammation in any part of the abdomen, a gouty habit, indigestible substances in the stomach, and the poison of lead.

"Another observation, says he, which it is desirable from its practical tendency to be acquainted with, is this: Spasm in one part may proceed from inflammation in another, and that which is calculated to lessen the original affection, is therefore best adapted to cure spasms of this description.

"In laying some stress upon this observation, I am actuated by the the recollection of cases, in which I have witnessed the most unsuccessful practice, from want, as I supposed, of this discrimination."

Several cases are given in confirmation of our author's opinions. The last subject treated in this practical and useful work is the connexion between disorders of the stomach and lungs. After shewing that consumption is frequently induced or accelerated by weakness of the stomach, he says, "In *all* cases of asthma, the stomach is disordered in its functions more or less; generally this disordered state of the stomach is a prelude to its approach; and whenever medical aid is successfully administered, the renovation of the powers of the stomach is the consequence of their operation.

"The attack of a paroxysm of periodic or convulsive asthma, (says Dr. Bree, in his work) is preceded very generally by dyspepsia, and the circumstances which occur to a relaxed habit. This condition of the body may have prevailed for months, or years, before it assumes the additional form of asthma; but when that disease is commenced, the symptoms of dyspepsia never fail to become aggravated, and to shew themselves with violence before the fit. These symptoms are flatulence, and distension of the stomach and bowels, a heavy pain over the forehead and eyes, eructation of wind with water, which is sometimes insipid, at others sour."

